# Neuro-cardiac imaging has a proven value in patient management: Con

**DOI:** 10.1007/s12350-017-0947-3

**Published:** 2017-06-07

**Authors:** Riccardo Liga, Arthur J. H. A. Scholte

**Affiliations:** 1 Cardio-Thoracic and Vascular Department, University Hospital of Pisa, Pisa, Italy; 2 Department of Cardiology, Leiden University Medical Center, PO Box 9600, 2300 RC Leiden, The Netherlands

## Introduction

The sympathetic nervous system (SNS) is important in cardiac pathophysiology. By increasing heart rate and arterial blood pressure, the SNS plays a central role to adapt cardiac performance to conditions of increased external needs.^1^ Accordingly, a sympathetic surge instantaneously ameliorates left ventricular (LV) contractility and relaxation, abruptly increasing cardiac working capacities. However, while an elevated sympathetic tone may be beneficial in the short term, a chronic elevation of SNS activity, as in the case of congestive heart failure (CHF), has been clearly associated with a significantly higher rate of adverse events.^2^,^3^ Accordingly, an accurate evaluation of cardiac SNS activity has become a *sine qua non* in modern clinical cardiology. However, most of the techniques for the assessment of cardiac sympathetic tone (i.e., baroreflex sensitivity) offer an indirect estimation of its level of activation.^4^ Nuclear cardiac imaging has been considered the reference standard for the evaluation of cardiac innervation, allowing the evaluation of SNS activity in (semi)-quantitative terms. Therefore, in the last decades, both positron emission tomography (PET)^5^ and conventional nuclear imaging techniques [either planar scintigraphy or single-photon emission computed tomography (SPECT)] have been demonstrated to allow the characterization of cardiac sympathetic innervation.^6^ On one hand, PET imaging, with its higher image resolution, radiotracers’ versatility, and intrinsically quantitative nature, is widely considered the quality standard of cardiac SNS evaluation. On the other hand, conventional nuclear imaging, with its robustness, availability, and relatively limited operational costs, still represents the most frequently performed imaging test for cardiac innervation imaging. More specifically, despite the theoretical advantages of more refined techniques (i.e., PET and SPECT), planar scintigraphy with ^123^I-metaiodobenzylguanidine (^123^I-MIBG) remains the workhorse of innervation imaging. Consequently, after almost 30 years from their introduction in the research arena, the two measures of cardiac sympathetic tone derived at planar scintigraphy, namely the heart-to-mediastinum (H/M) ratio and the washout rate of ^123^I-MIBG, still represent the most validated measures of LV innervation that are available, with the H/M ratio as reference standard.^7^ So, a depressed H/M ratio of ^123^I-MIBG has been shown to associate with a significantly worse prognosis, independently of LV ejection fraction.^3^ Moreover, in those years, a plethora of small-numbered studies have demonstrated the association between abnormal H/M values and various measures of LV structural and functional impairment, almost translating it as a *universal* marker of cardiac risk. Nevertheless, despite these apparently promising results, cardiac innervation imaging has a minimal, if any, clinical role in western countries, with currently no indications in major clinical guidelines, except for Japan.

In order to understand this, some of the most evident limitations of cardiac ^123^I-MIBG imaging should be carefully considered.

### Standardization of Protocol

The scan protocol that is used for ^123^I-MIBG planar imaging varies widely, with the delayed acquisition—the clinically relevant one—more often performed 4 hours after tracer injection.^7^ However, this rather lengthy imaging protocol ends up consistently affecting patients' compliance, reducing the possible throughput of the technique, and discouraging the performance of additional, albeit time-consuming, dedicated cardiac acquisitions (i.e., SPECT when performed with traditional Anger cameras).

Interestingly, the specifics of this apparently standardized protocol are based more on habits than on experimental proof. In fact, solid evidence has demonstrated that the real kinetics of ^123^I-MIBG in human hearts is consistently more rapid than initially hypothesized,^8^ theoretically allowing a significantly faster image protocol, with the late scan performed even 1 hour after tracer administration.^9^

### Instrumentation

It has been demonstrated that, due to the specific emission spectrum of ^123^I-photons, medium-energy collimators should be better used for cardiac ^123^I-MIBG imaging, in order to obtain both high-quality images and accurate estimates of the H/M ratio.^10^ However, the majority of the diagnostic and prognostic data regarding ^123^I-MIBG imaging, including the ones coming from the ADMIRE-HF study,^3^ have been obtained with low-energy collimators, making the validity of the results obtained somehow questionable.

For similar reasons, no clinically useful cut-off value of H/M ratio has yet been proposed, since the most frequently used normalcy value of 1.6 (derived from 202 historical controls gathered from 7 studies)^11^ has been only validated in patients with CHF, demonstrating, anyway, an unsatisfactory diagnostic accuracy as a biomarker of cardiac risk.

The elevated methodological inhomogeneity of ^123^I-MIBG reports is even more evident when studies performed, for instance, in Japan and Europe/US are compared, with the former often using different instrumentation and scanning protocols (i.e., medium-energy collimators). These technical disparities may allow to question the generalizability of the many analysis of pooled ^123^I-MIBG data derived from studies performed in population of patients with different ethnicities (Caucasian vs Asiatic) and imaged in different scanning conditions.^12^

All together, these basic technical disadvantages of planar ^123^I-MIBG imaging allow to question the role of cardiac innervation parameters as clinically useful biomarkers of cardiac risk. While more elegant imaging techniques are available—SPECT and PET—they are even less validated than planar imaging and are rarely employed for clinical purposes. According to the authors’ beliefs, a redefinition of the role of cardiac sympathetic innervation imaging is eagerly needed, clearly identifying the methodology to be used and the specific fields of application.

In this respect, the most relevant limitations of current cardiac innervation imaging modalities in specific clinical settings (i.e., CHF and ventricular arrhythmias) will be further discussed.

## Cardiac Innervation Imaging in Heart Failure: Chances and Limits

SNS plays a central role in the genesis and maintenance of CHF. In fact, evidence has conclusively demonstrated that an increased SNS activity is not only associated with the presence of CHF, but rather represent one of its most prominent (con)-causative factors.^4^ In the last decades, nuclear cardiac imaging has allowed the characterization of the interaction between sympathetic dysregulation and abnormal LV function, showing that patients with CHF typically present a condition of SNS hyper-activation coupled with post-synaptic myocardial sympathetic desensitization, due to reduced beta-adrenoceptor density and catecholamine spill-over.^3^,^13^ In this respect, despite a number of small mechanistic studies have suggested the possible value of cardiac innervation imaging in the risk stratification and management of patients with CHF, the most solid evidence in favor of its application in clinical practice comes from the ADMIRE-HF study.^3^ By enrolling nearly one thousand subjects with depressed LV ejection fraction and moderately symptomatic CHF (NYHA class II-III) of mixed etiology (66% ischemic and 34% non-ischemic), the ADMIRE-HF study demonstrated that the H/M ratio of ^123^I-MIBG was able to risk-stratify patients independently of other commonly measured cardiac functional parameters, such as LV ejection fraction and BNP. However, in order to be introduced in clinical practice, any biomarker must have a number of necessary qualities that should be always taken into account: a cut-off value characterized by a relevant accuracy in unmasking high-risk patients, the clear definition of the categories of patients where it should be applied, and the demonstration of its significant impact on patient management in terms of either prognosis or cost-benefit. Accordingly, despite the encouraging results coming from ADMIRE-HF, relevant limitations of the studies performed so far have prevented the clinical application of cardiac innervation imaging. First of all, no real cut-off of H/M ratio actually exists. In fact, the H/M ratio is rather characterized by a continuous association with patients risk^14^ and the cut-off values proposed so far have only shown a modest diagnostic accuracy in discriminating patients' risk categories.^15^ Moreover, while the ADMIRE-HF trial was designed to include only patients with severely compromised LV systolic function, it was demonstrated that 43% of them had a LV ejection fraction >35% at core-lab analysis,^16^ making the main results of the study rather questionable and a clear definition of the category of patients that may benefit from innervation imaging still lacking.

However, the prominent limitation of the existing literature regarding cardiac SNS imaging is that it lacks a prospective evaluation of the prognostic impact of innervation parameters on clinical decision-making and patient management. In fact, as in the case of any biomarker, it should be determined whether measures of cardiac sympathetic tone may be used to guide and monitor therapeutic interventions, helping the clinician to prognosticate the result of specific therapies and to decide, for instance, on the non-use of an otherwise indicated strategies. To date, only few studies performed in patients mainly suffering from CHF have evaluated how cardiac sympathetic innervation parameters might predict patient response to selected cardioactive treatments, suggesting the existence of some correlation between measures of innervation at baseline and the effect of the therapies.^17^,^18^ However, most of those reports did not assess true patient outcomes but rather surrogate endpoint of treatment efficacy (i.e., change in LV ejection fraction)^17^ and were not randomizing patients to strategies with and without innervation imaging.^18^

After all those years, all the different disease-modifying therapeutic strategies that can be adopted in patients with CHF are only selected according to the presence of classical cardiac structural and functional abnormalities (i.e., ECG abnormalities or a depressed LV ejection fraction), disregarding any parameter of sympathetic innervation. In other words, despite the existing evidence, no one would ever exclude any patient with CHF from a given treatment, either pharmacological or device-based [i.e., cardiac resynchronization therapy (CRT)], based on the findings of cardiac innervation imaging, such as a depressed H/M ratio of ^123^I-MIBG. More innovative imaging techniques, such as SPECT acquisition particularly if performed with dedicated CZT devices, might allow to overcome some of the limitations of planar ^123^I-MIBG scans, giving the opportunity to obtain a three-dimensional measures of cardiac sympathetic tone despite a contained acquisition time.^18^ However, a reliable prospective validation of the added value of such imaging modalities in the management of CHF is eagerly awaited.

## Innervation Imaging in Patients with IHD and Increased Cardiac Risk

Sympathetic nervous terminals are significantly more sensitive to ischemia than myocardial cells. In fact, after an acute myocardial infarction, the area of LV sympathetic denervation typically exceeds the extent of myocardial necrosis.^19^ Thereafter, during the recovery phase, autonomic nerve terminals sprout from the borderzone of the infarction with progressive cardiac reinnervation. However, the healing process is frequently incoherent, leading to the formation of islands of viable but denervated myocardium surrounded by hyper-innervated regions that ultimately represent possible sites of origin of malignant ventricular arrhythmias.^20^

Accordingly, it is not surprising that measures of cardiac sympathetic denervation have been repeatedly associated with an increased risk of arrhythmic events, possibly helping to better risk-stratify cardiac patients. Specifically, results coming from the ADMIRE-HF study have shown that patients with a H/M ratio <1.6 had a significantly higher incidence of malignant ventricular arrhythmic episodes, even fatal, than those with a relatively preserved cardiac sympathetic tone.^3^ However, despite some initially encouraging data, ^123^I-MIBG scintigraphy has proved to be a rather inaccurate method for the prediction of malignant ventricular arrhythmias, since patients with a preserved innervation (i.e., H/M ratio >1.6) still have a significant incidence of arrhythmic events or sudden cardiac deaths (roughly 3% in the ADMIRE-HF cohort), and does not select patients that may have improved survival with implantable cardioverter defibrillator (ICD) implantation.^17^ Therefore, for many categories of patients, the presence of a significantly depressed LV ejection fraction (i.e., less than 30%-35%) is frequently considered the only indication for ICD therapy.

Interestingly, after many years, the currently on-going ADMIRE-ICD study (NCT02656329) will be the first randomized trial to evaluate the efficacy of ^123^I-MIBG imaging for guiding the decision of ICD implantation. However, since this study will only enroll patients with a narrow range of EF values (between 30% and 35%), it could, curiously, further limit the categories of patients that could benefit from cardiac innervation imaging.

In this scenario, planar scintigraphy badly adapts to appropriately describe the patterns of cardiac sympathetic denervation in patients with IHD and post-ischemic LV dysfunction who frequently show areas of viable myocardium interposed within scarred regions. In fact, limited evidence has suggested that the use of tomographic imaging techniques, such as SPECT or PET, would allow an accurate depiction of the regional distribution of sympathetic nerve terminals and a better risk stratification than traditional planar measures.^19^ Moreover, by integrating information coming also from the evaluation of myocardial perfusion data, the innervation imaging of the future will probably allow the combined assessment of two of the most relevant cardiac functional parameters and the accurate localization of the areas of viable myocardium showing a depressed sympathetic innervation. In fact, the presence of those myocardial regions showing an “innervation/perfusion mismatch” (i.e., at the border zone of myocardial scars), representing preferred sites of origin of malignant ventricular arrhythmias,^20^ has been clearly associated with adverse patients' prognosis.^19^ An elevated regional burden of jeopardized myocardium with impaired sympathetic innervation may possibly represent an additional marker of risk in patients with IHD, possibly predicting the need for aggressive therapeutic strategies.^19^,^20^ Nevertheless, while the possible clinical impact of those innovative parameters of regional sympathetic denervation has been recently suggested by some small studies, an appropriate validation of this diagnostic approach is still awaited and the current clinical role of innervation imaging in the management of patients at risk of malignant ventricular arrhythmias almost inexistent.

## Conclusion

Nuclear cardiac imaging is the only accurate tool for the assessment of the interaction between LV sympathetic innervation and cardiac function. Among the different methodologies that have been proposed, a rather rudimentary technique, namely planar ^123^I-MIBG scintigraphy, still remains the state-of-the-art for the evaluation of cardiac sympathetic innervation. However, while evidence has been accumulating on the possible role of innervation imaging for the risk stratification and disease management of cardiac patients, none of these techniques has never reached the clinical arena and is mainly considered a purely research investigation. A number of reasons may justify this apparent conundrum. First of all, large trials focused on specific categories of patients to clearly identify the possible fields of applications of cardiac innervation imaging (i.e., management of patients with borderline indication for ICD implantation or selection of patients with malignant ventricular arrhythmias undergoing invasive procedures) are still lacking. On the other hand, the panel of clinically available nuclear techniques should be further implemented, with the progressive abandonment of “old-fashioned” methodologies and measures (i.e., H/M ratio of ^123^I-MIBG), and the introduction of innovative markers of risk obtained with novel technologies (i.e., CZT cameras), such as the regional extent of LV sympathetic denervation or the burden of innervation/perfusion mismatch.

Otherwise, it is conceivable that nuclear innervation imaging will remain one of the “holy grails” of modern cardiology, with a solid pathophysiologic background but limited, if any, clinical applications and patients will be further treated according to other biomarkers, such as LV ejection fraction.

## References

[1] Triposkiadis F, Karayannis G, Giamouzis G, Skoularigis J, Louridas G, Butler J (2009). The sympathetic nervous system in heart failure physiology, pathophysiology, and clinical implications. J Am Coll Cardiol.

[2] Eisenhofer G, Friberg P, Rundqvist B, Quyyumi AA, Lambert G, Kaye DM (1996). Cardiac sympathetic nerve function in congestive heart failure. Circulation..

[3] Jacobson AF, Senior R, Cerqueira MD, Wong ND, Thomas GS, Lopez VA (2010). Myocardial iodine-123 meta-iodobenzylguanidine imaging and cardiac events in heart failure. Results of the prospective ADMIRE-HF (AdreView Myocardial Imaging for Risk Evaluation in Heart Failure) study. J Am Coll Cardiol.

[4] Florea VG, Cohn JN (2014). The autonomic nervous system and heart failure. Circ Res..

[5] Thackeray JT, Bengel FM (2013). Assessment of cardiac autonomic neuronal function using PET imaging. J Nucl Cardiol..

[6] Travin MI (2013). Cardiac autonomic imaging with SPECT tracers. J Nucl Cardiol..

[7] Flotats A, Carrió I, Agostini D, Le Guludec D, Marcassa C, Schäfers M (2010). Proposal for standardization of 123I-metaiodobenzylguanidine (MIBG) cardiac sympathetic imaging by the EANM Cardiovascular Committee and the European Council of Nuclear Cardiology. Eur J Nucl Med Mol Imaging..

[8] Tinti E, Positano V, Giorgetti A, Marzullo P (2014). Feasibility of [(123)I]-meta-iodobenzylguanidine dynamic 3-D kinetic analysis in vivo using a CZT ultrafast camera: Preliminary results. Eur J Nucl Med Mol Imaging..

[9] Dimitriu-Leen AC, Gimelli A, Al Younis I, Veltman CE, Verberne HJ, Wolterbeek R (2016). The impact of acquisition time of planar cardiac (123)I-MIBG imaging on the late heart to mediastinum ratio. Eur J Nucl Med Mol Imaging..

[10] Inoue Y, Abe Y, Kikuchi K, Matsunaga K, Masuda R, Nishiyama K (2016). Correction of collimator-dependent differences in the heart-to-mediastinum ratio in ^123^I-metaiodobenzylguanidine cardiac sympathetic imaging: Determination of conversion equations using point-source imaging. J Nucl Cardiol..

[11] Jacobson AF, Lombard J, Banerjee G, Camici PG (2009). 123I-mIBG scintigraphy to predict risk for adverse cardiac outcomes in heart failure patients: Design of two prospective multicenter international trials. J Nucl Cardiol..

[12] Verberne HJ, Brewster LM, Somsen GA, van Eck-Smit BL (2008). Prognostic value of myocardial 123I-metaiodobenzylguanidine (MIBG) parameters in patients with heart failure: A systematic review. Eur Heart J..

[13] Gaemperli O, Liga R, Spyrou N, Rosen SD, Foale R, Kooner JS (2010). Myocardial beta-adrenoceptor down-regulation early after infarction is associated with long-term incidence of congestive heart failure. Eur Heart J..

[14] Verschure DO, Veltman CE, Manrique A, Somsen GA, Koutelou M, Katsikis A (2014). For what endpoint does myocardial 123I-MIBG scintigraphy have the greatest prognostic value in patients with chronic heart failure? Results of a pooled individual patient data meta-analysis. Eur Heart J Cardiovasc Imaging..

[15] Shah AM, Bourgoun M, Narula J, Jacobson AF, Solomon SD (2012). Influence of ejection fraction on the prognostic value of sympathetic innervation imaging with iodine-123 MIBG in heart failure. JACC Cardiovasc Imaging..

[16] Choi JY, Lee KH, Hong KP, Kim BT, Seo JD, Lee WR (2001). Iodine-123 MIBG imaging before treatment of heart failure with carvedilol to predict improvement of left ventricular function and exercise capacity. J Nucl Cardiol..

[17] Hachamovitch R, Nutter B, Menon V, Cerqueira MD (2015). Predicting risk versus predicting potential survival benefit using 123I-mIBG imaging in patients with systolic dysfunction eligible for implantable cardiac defibrillator implantation: Analysis of data from the prospective ADMIRE-HF study. Circ Cardiovasc Imaging..

[18] Gimelli A, Liga R, Giorgetti A, Genovesi D, Marzullo P (2014). Assessment of myocardial adrenergic innervation with a solid-state dedicated cardiac cadmium-zinc-telluride camera: First clinical experience. Eur Heart J Cardiovasc Imaging..

[19] Fallavollita JA, Heavey BM, Luisi AJ, Michalek SM, Baldwa S, Mashtare TL (2014). Regional myocardial sympathetic denervation predicts the risk of sudden cardiac arrest in ischemic cardiomyopathy. J Am Coll Cardiol.

[20] Gimelli A, Menichetti F, Soldati E, Liga R, Vannozzi A, Marzullo P (2016). Relationships between cardiac innervation/perfusion imbalance and ventricular arrhythmias: Impact on invasive electrophysiological parameters and ablation procedures. Eur J Nucl Med Mol Imaging..

